# Beyond desensitization: the SCFA-Treg-gut microbiota axis as a mechanistic framework for sustained unresponsiveness in food allergy

**DOI:** 10.3389/fimmu.2026.1949158

**Published:** 2026-09-14

**Authors:** Chao Song, Qingyu Zhang, Zhen Li, Hong Zhang, Zhengtao Chen, Qinghua Liu

**Affiliations:** 1 Department of Clinical Laboratory, The Second Affiliated Hospital of Shandong First Medical University, Tai’an, China; 2 Department of Hematology, The Second Affiliated Hospital of Shandong First Medical University, Tai’an, China; 3 School of Laboratory Animal Science, Shandong First Medical University & Shandong Academy of Medical Sciences, Jinan, China

**Keywords:** food allergy, gut microbiota, oral immunotherapy, regulatory T cells, remission, short-chain fatty acids, sustained unresponsiveness

## Abstract

Food allergy is an immune-mediated condition associated with acute reactions, risk of anaphylaxis, and a substantial day-to-day management burden. Oral immunotherapy (OIT) can increase the amount of allergen tolerated during treatment, but this desensitized state commonly depends on continued exposure. Sustained unresponsiveness (SU), also termed remission in some trials, is a stricter operational endpoint in which clinical non-reactivity is retained after a predefined period of treatment withdrawal and is confirmed by oral food challenge. The biological processes that determine whether desensitization develops into SU remain incompletely understood. This narrative review evaluates the gut microbiota-short-chain fatty acid (SCFA)-regulatory T-cell (Treg) axis as a mechanistic framework for this transition. Experimental studies show that microbial SCFAs can influence Treg differentiation and function through histone deacetylase inhibition and metabolite-sensing receptors, while also conditioning epithelial and antigen-presenting-cell programs that favor mucosal tolerance. Human cohort and microbiota-transfer studies support an association between early-life microbial maturation, SCFA-related metabolic capacity, and allergic outcomes, but most causal evidence remains preclinical. Importantly, fecal SCFA concentrations are imperfect surrogates for mucosal exposure, and a randomized trial of oral butyrate added to peanut OIT did not improve SU. These findings argue against treating a single metabolite as a sufficient therapeutic target. Translational development should instead test spatially targeted metabolite delivery, defined microbial therapeutics, and precision prebiotic or synbiotic strategies as adjuncts to antigen-directed therapy. Future trials require harmonized post-withdrawal challenge definitions, longitudinal sampling of microbial function and host immunity, and biomarker-guided patient stratification. The SCFA-Treg-gut microbiota axis is therefore best viewed as a testable, integrative model of durable immune control rather than an established therapeutic pathway to SU.

## 1 Introduction

### 1.1 Treatment goals in food allergy: from risk reduction to durable immune control

Food allergy affects children and adults and imposes a burden that extends beyond the acute risk of allergic reactions. Patients and caregivers must maintain allergen avoidance, interpret food labels, coordinate school and family supervision, carry rescue medication, and manage persistent uncertainty about accidental exposure (1, 2). Current management therefore combines accurate diagnosis, individualized avoidance and nutritional advice, emergency education, and access to intramuscular epinephrine for patients at risk of anaphylaxis (3). These measures remain essential, but they primarily reduce exposure-related risk rather than directly modifying allergen-specific immune reactivity.

Oral immunotherapy (OIT) is the most extensively studied active treatment for immunoglobulin E (IgE)-mediated food allergy and is recommended or suggested for selected allergens and age groups in contemporary guidelines (3, 4). Its established clinical effect is desensitization: repeated, controlled allergen ingestion raises the reaction threshold while treatment continues. OIT is accompanied by changes in allergen-specific IgE and immunoglobulin G4 (IgG4), basophil and mast-cell responsiveness, and allergen-reactive T-cell programs (5). However, the achieved threshold can decline when daily dosing is reduced or discontinued, as demonstrated in the POISED study and other withdrawal designs (6, 7). Thus, the central translational question is not only whether OIT protects against low-dose accidental exposure during treatment, but also why durable post-treatment non-reactivity develops in only a subset of patients.

### 1.2 Desensitization, sustained unresponsiveness, and remission are related but non-equivalent endpoints

Desensitization denotes an increased reaction threshold during ongoing allergen exposure or maintenance therapy. SU is an operational research endpoint assessed after treatment cessation and a prespecified avoidance interval, followed by an oral food challenge. Some recent trials use the term remission to emphasize that post-treatment non-reactivity is neither equivalent to natural tolerance nor proven to be permanent (5–7). The terms are therefore not fully interchangeable across studies, because withdrawal duration, challenge dose, permitted background ingestion, and follow-up time differ substantially.

These endpoint differences directly affect interpretation. A participant who passes an end-of-treatment challenge but reacts after withdrawal has achieved desensitization without SU. In contrast, a participant who remains non-reactive after withdrawal has met the protocol-specific SU or remission definition, but still requires longer follow-up to establish durability. Age, baseline allergen-specific IgE, treatment duration, dose, and immune phenotype have all been associated with post-treatment outcomes, yet no single marker reliably defines a stable tolerant state (6, 7). Accordingly, future studies should report the exact withdrawal interval, challenge threshold, regular ingestion after challenge, and longer-term clinical status rather than treating SU as a uniform binary outcome.

### 1.3 Scope and review approach

Short-chain fatty acids (SCFAs) are microbial fermentation products, principally acetate, propionate, and butyrate, generated from nondigestible dietary substrates, whereas regulatory T cells (Tregs) are forkhead box P3 (FOXP3)-expressing CD4-positive T cells that constrain inappropriate immune activation and contribute to mucosal tolerance (10–13).

This narrative review uses the SCFA-Treg-gut microbiota axis to organize evidence linking microbial ecology, metabolite exposure, epithelial barrier function, antigen presentation, and regulatory immunity. Targeted searches of PubMed and ClinicalTrials.gov were updated through July 2026 using combinations of “food allergy,” “oral immunotherapy,” “sustained unresponsiveness,” “remission,” “microbiota,” “short-chain fatty acid,” “butyrate,” “regulatory T cell,” and “fecal microbiota transplantation.” Human interventional studies and longitudinal cohorts were prioritized for translational claims, while landmark mechanistic studies were used to define biological plausibility. Because this was not a systematic review, the discussion emphasizes evidence hierarchy, mechanistic consistency, and limits of inference rather than quantitative effect estimation.

The axis is not presented as the sole explanation for food allergy or SU. Instead, it provides an integrative model in which food antigen is encountered within a local eco-immunological context. Microbial metabolites may influence whether epithelial and antigen-presenting cells (APCs) transmit danger or regulatory signals, and whether Tregs maintain lineage stability under allergic inflammation (8, 9). This framework helps distinguish the antigen-specific effects of OIT from the broader mucosal conditions that may support or undermine post-withdrawal immune control.

Previous reviews have extensively discussed the gut microbiome in food allergy, microbial metabolites in oral tolerance, and nutritional or microbiota-targeted strategies (8, 9, 14–16). The present review is organized around a narrower translational question: whether the microbiota-SCFA-Treg network can help explain the transition from exposure-dependent OIT desensitization to post-withdrawal SU. Accordingly, we distinguish evidence supporting allergic sensitization or oral tolerance from evidence directly addressing OIT and SU, and we identify relationships that remain extrapolative or hypothesis-generating. The overall conceptual framework is summarized in **Figure 1**.

**Figure 1:**
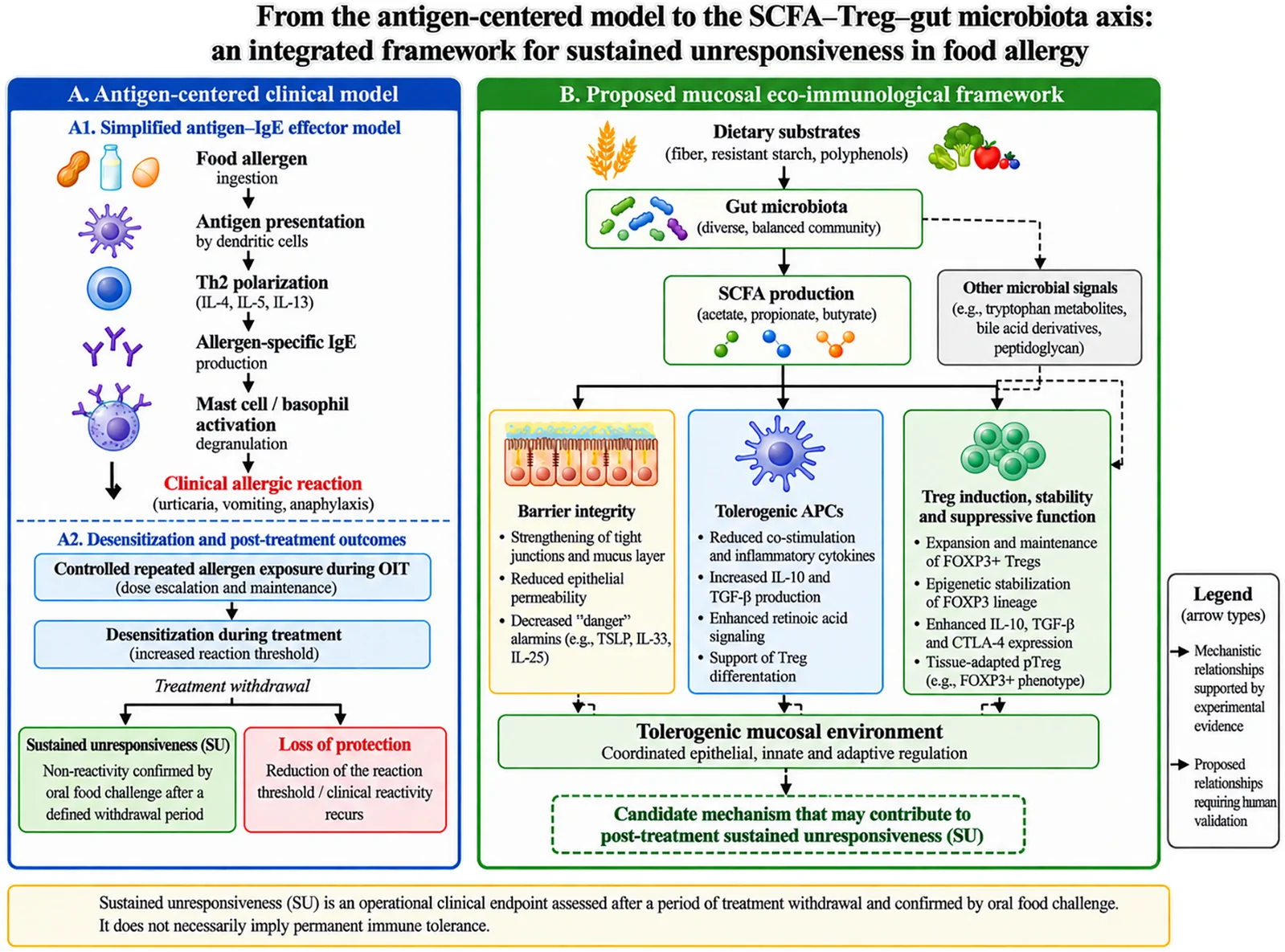
From the antigen-centered model to the SCFA–Treg–gut microbiota axis: an integrated framework for sustained unresponsiveness in food allergy. **(A)** The simplified antigen-centered clinical model depicts the sequence from food-allergen ingestion and antigen presentation to Th2 polarization, allergen-specific IgE production, mast-cell and basophil activation, and clinical allergic reactions. Controlled repeated allergen exposure during oral immunotherapy (OIT) may increase the reaction threshold and induce desensitization while treatment is continued. After treatment withdrawal, patients may either maintain clinical non-reactivity, operationally defined as sustained unresponsiveness (SU), or experience a decline in the reaction threshold and recurrence of clinical reactivity. **(B)** The proposed mucosal eco-immunological framework incorporates dietary substrates, the gut microbiota, short-chain fatty acid (SCFA) production, and other microbial signals. These factors may coordinately influence epithelial-barrier integrity, tolerogenic antigen-presenting-cell conditioning, and regulatory T-cell induction, lineage stability, and suppressive function, thereby supporting a tolerogenic mucosal environment. Solid arrows indicate mechanistic or clinical relationships supported by experimental evidence, whereas dashed arrows indicate proposed relationships that require further validation in humans. SU is an operational post-treatment clinical endpoint assessed after a defined period of treatment withdrawal and does not necessarily imply absolute or permanent immune tolerance. pTreg, peripherally induced regulatory T cell.

## 2 Mechanistic basis of the SCFA-Treg-gut microbiota axis

### 2.1 Dietary substrates, microbial fermentation, and biologically relevant SCFA exposure

Acetate, propionate, and butyrate are the major SCFAs generated by anaerobic fermentation of nondigestible carbohydrates. Fiber structure, physicochemical properties, fermentation rate, and access by primary degraders influence which substrates enter microbial fermentation (10, 17, 18). Community metabolism then depends on cross-feeding: acetate or other fermentation products released by one organism can be used by another, and several Roseburia, Faecalibacterium, Eubacterium, and Anaerostipes lineages contribute to butyrate formation through partially overlapping pathways (17, 18). Propionate can arise through succinate, acrylate, and propanediol pathways, whose distribution is taxon- and substrate-dependent (18). This functional redundancy means that taxonomic composition alone, including the abundance of a single presumed beneficial genus, cannot reliably predict net SCFA output.

Fecal SCFA concentrations reflect the residual pool after microbial production, epithelial uptake, host metabolism, and intestinal transit (10, 19). Butyrate is extensively used by colonocytes, propionate is substantially extracted by the liver, and acetate more readily reaches the peripheral circulation (10). Consequently, stool concentrations do not directly measure SCFA exposure at the epithelial surface or within the lamina propria. A low fecal concentration may reflect low production or efficient uptake, whereas a high value may reflect high production, altered absorption, or transit. For food-allergy research, the amount, anatomical site, timing, and cellular availability of SCFAs are therefore more informative than stool concentration alone (see Section 4.2).

### 2.2 Direct effects of SCFAs on Treg differentiation, stability, and suppressive function

Landmark mouse studies established that SCFAs can expand or induce colonic FOXP3-positive Tregs (11–13). Butyrate and propionate inhibit histone deacetylases and increase histone acetylation at regulatory regions associated with FOXP3 expression, while SCFA signaling through free fatty acid receptor 2 (FFAR2, also known as GPR43) contributes to the size and function of the colonic Treg pool (11–13). These findings support a biologically plausible route from microbial fermentation to regulatory immunity. Nevertheless, the magnitude, tissue specificity, and dose-response relationships demonstrated in mice cannot be assumed to occur identically in food-allergic patients.

Commensal Clostridia can promote colonic Treg accumulation, and defined mixtures of human-derived Clostridia strains induce regulatory programs in germ-free mice (20, 21). Microbiota-dependent retinoic acid receptor-related orphan receptor γt (RORγt)-positive peripheral Tregs are particularly relevant to intestinal immune homeostasis and suppression of type 2 responses. In the context of food allergy, however, RORγt-positive Tregs should be described as a strongly supported experimental mechanism rather than a validated clinical biomarker. Peripheral-blood Treg frequency alone is unlikely to capture the tissue localization, clonality, lineage stability, and suppressive competence that determine mucosal function.

SCFA signaling is receptor- and context-dependent. Acetate and propionate are prominent ligands for FFAR2/GPR43, whereas propionate and butyrate can also activate free fatty acid receptor 3 (FFAR3, also known as GPR41); butyrate additionally signals through hydroxycarboxylic acid receptor 2 (HCAR2, also known as GPR109A) (22, 23). These receptor-mediated effects coexist with receptor-independent histone deacetylase inhibition, particularly for butyrate and propionate (11–13).

Tregs restrain allergic and mucosal immune responses through multiple effector mechanisms. Cytotoxic T-lymphocyte-associated protein 4 (CTLA-4) can reduce antigen-presenting cell costimulatory capacity by removing CD80/CD86 from the cell surface, thereby limiting CD28-dependent activation (24). Interleukin-10 (IL-10) and transforming growth factor β (TGF-β) contribute to suppression of inflammatory signaling and maintenance of a low-reactivity mucosal environment, while FOXP3-positive regulatory populations can shape mucosal immunoglobulin A (IgA) selection and host-microbiota homeostasis (14, 25, 26). Tregs can also directly dampen IgE-dependent mast-cell degranulation through OX40-OX40 ligand (OX40L) interactions in experimental systems (27). These functions provide a mechanistic bridge between regulatory phenotype and effector restraint, but restoration of tolerance cannot be inferred from a numerical increase in circulating Tregs alone; lineage stability, tissue localization, allergen specificity, and functional suppression are more informative.

### 2.3 Indirect effects of SCFAs on antigen-presenting cells and the epithelial barrier

SCFAs also act on non-T-cell compartments. In experimental systems, GPR109A signaling promotes anti-inflammatory properties in colonic macrophages and dendritic cells and supports induction of IL-10-producing and regulatory T cells (22). GPR43 and GPR109A signaling in non-hematopoietic cells can also support epithelial homeostasis (28). In a mouse model of food allergy, dietary fiber and SCFAs enhanced retinaldehyde dehydrogenase activity in CD103-positive dendritic cells, increased regulatory pathways, and reduced allergic responses (29). These studies link microbial metabolism to the antigen-presenting context in which food proteins are encountered.

The epithelial barrier regulates antigen access and produces signals that shape type 2 immunity. Thymic stromal lymphopoietin (TSLP), interleukin-33 (IL-33), and interleukin-25 (IL-25) can amplify group 2 innate lymphoid cell (ILC2)- and type 2 helper T-cell (Th2)-associated inflammation when the epithelium is injured; food-allergy mouse studies support roles for these alarmins, although their relative importance varies by model (30, 31). SCFAs may support epithelial energy metabolism, mucus production, tight-junction regulation, and barrier repair, but much of this evidence derives from colitis or general intestinal-homeostasis models rather than human food allergy (28–30, 32). The axis should therefore be understood as a network of interactions with different levels of direct relevance to the allergic mucosa. These cellular interfaces are summarized in **Figure 2**.

**Figure 2:**
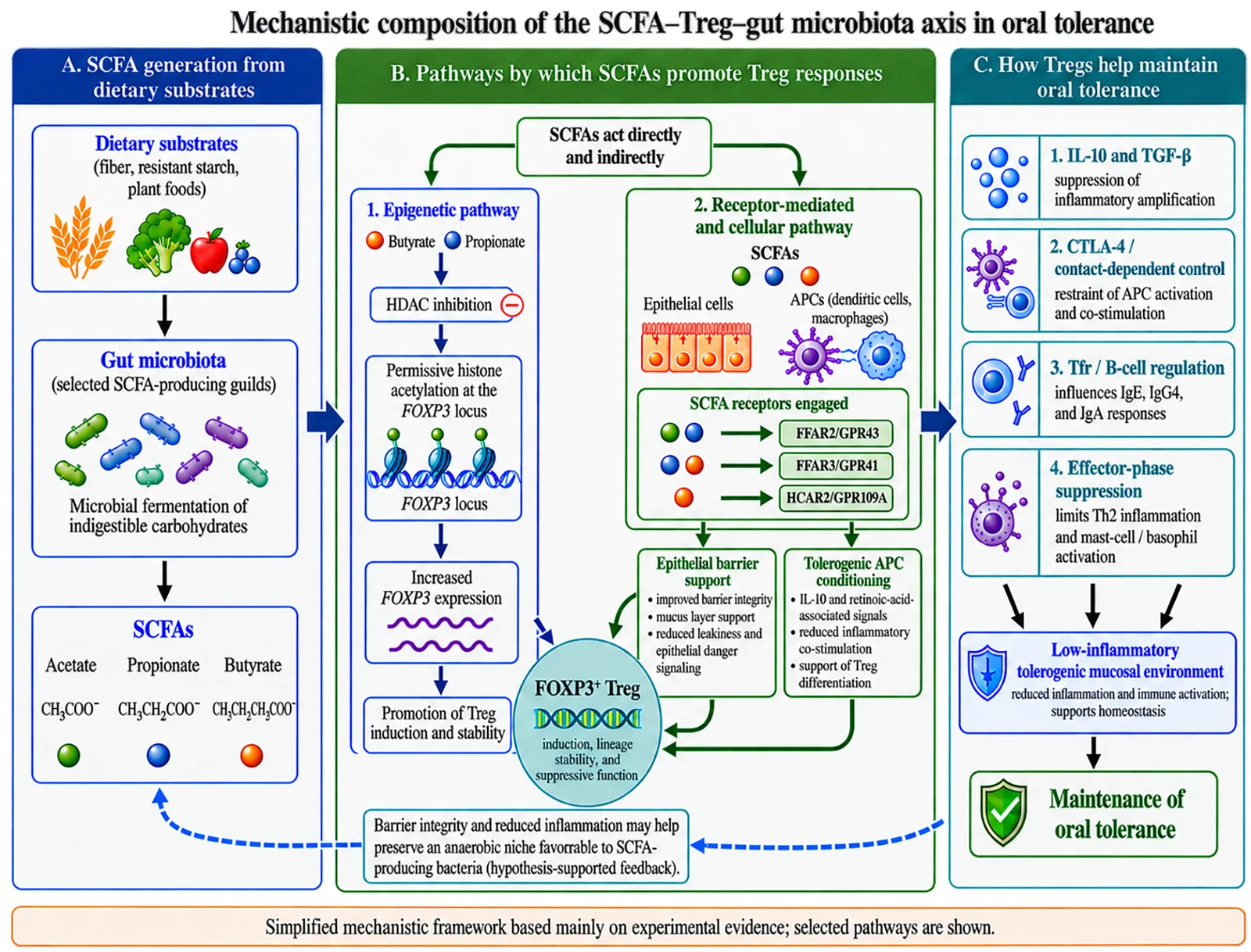
Mechanistic composition of the SCFA–Treg–gut microbiota axis in oral tolerance. **(A)** Dietary fibers, resistant starches, and other fermentable plant-derived substrates are metabolized by selected gut microbial communities to generate the principal short-chain fatty acids (SCFAs), acetate, propionate, and butyrate. **(B)** SCFAs may promote regulatory T-cell responses through complementary direct and indirect pathways. In the epigenetic pathway, butyrate and propionate inhibit histone deacetylases (HDACs), promoting permissive histone acetylation at regulatory regions of the FOXP3 locus and thereby supporting FOXP3 expression, Treg induction, and lineage stability. In parallel, SCFAs engage metabolite-sensing receptors, including FFAR2/GPR43, FFAR3/GPR41, and HCAR2/GPR109A, and may condition epithelial cells and antigen-presenting cells toward barrier-supportive and tolerogenic phenotypes. These pathways converge on the induction, stability, and suppressive function of FOXP3-positive Tregs. Barrier integrity and reduced inflammation may, in turn, help preserve an anaerobic intestinal niche favorable to SCFA-producing microorganisms; this feedback relationship remains hypothesis-supported and requires further human validation. **(C)** Tregs contribute to oral tolerance through IL-10- and TGF-β-mediated suppression of inflammatory amplification, CTLA-4-dependent restraint of antigen-presenting-cell activation and co-stimulation, regulation of B-cell and immunoglobulin responses by follicular regulatory T cells, and suppression of Th2 inflammation and mast-cell or basophil activation during the effector phase. Together, these mechanisms support a low-inflammatory tolerogenic mucosal environment and maintenance of oral tolerance. The figure represents a simplified mechanistic framework based predominantly on experimental evidence, and selected pathways are shown. Tfr cells, follicular regulatory T cells.

## 3 How the axis may fail in food allergy

### 3.1 Altered microbiota maturation and fermentative capacity

Early life is a critical period during which microbial communities, epithelial function, and mucosal immunity mature together. Cesarean delivery, antibiotic exposure, feeding practices, diet, and environmental microbial contact are associated with differences in infant microbiota development, although these exposures should not be treated as deterministic causes of food allergy (8, 9). The more relevant question is whether altered microbial maturation changes functional outputs required for barrier and regulatory immune development.

Human-to-mouse microbiota-transfer studies provide causal evidence within experimental systems. Germ-free mice colonized with microbiota from healthy infants were protected from cow’s-milk-allergen responses, whereas microbiota from affected infants did not confer equivalent protection (33). Separate work showed that defined bacterial therapy suppressed food allergy through a Treg myeloid differentiation primary response 88 (MyD88)/RORγt pathway in mice (34). Human cohort data further associate high fecal butyrate and propionate in infancy with lower subsequent atopy and reported food allergy (35). Together, these studies support a developmental association and experimental mechanism, but they do not establish that a single taxon or fecal metabolite concentration is sufficient to predict or prevent food allergy in humans.

Human studies nevertheless indicate that food allergy is associated with alterations in gut microbial ecology, although no single reproducible taxonomic signature has emerged. In one prospective cohort, Haemophilus, Dialister, Dorea, and Clostridium were underrepresented in infancy among children who later developed food sensitization, whereas Citrobacter, Oscillospira, Lactococcus, and Dorea were underrepresented among those who developed food allergy (36). More recently, two Japanese birth cohorts identified distinct one-month enterotypes: a Bifidobacterium-dominant enterotype, characterized by higher fecal propionate, was associated with lower subsequent risks of food sensitization and food allergy than Bacteroides- or Klebsiella-dominant enterotypes (37). Together with the microbiota-transfer findings from affected infants described above (33), these observations support alterations in microbial ecology associated with food allergy and its development, while also showing that the specific taxonomic patterns vary across populations, age groups, dietary backgrounds, and study designs. Accordingly, current evidence does not support a universal “protective” or “risk” microbiome defined by a small number of taxa. For the proposed axis, community-level capacity to use fermentable substrates and sustain cross-feeding networks that generate SCFAs may therefore be more informative than the abundance of any individual genus (15, 17, 18). Altered early-life microbial maturation could therefore impair the axis by changing local SCFA availability, which may in turn affect epithelial conditioning and mucosal Treg development; in humans, this sequence remains a mechanistic hypothesis rather than an established causal chain.

### 3.2 Barrier dysfunction and epithelial danger signaling

Food antigen does not act in isolation. Barrier disruption can increase antigen access to the lamina propria, while epithelial alarmins such as TSLP, IL-33, and IL-25 can promote type 2 immune programs; food-allergy mouse studies support roles for these signals, although their relative contribution is model-dependent (30, 31). A second, ecological step has weaker food-allergy-specific support. In broader intestinal homeostasis and inflammatory models, loss of butyrate-dependent epithelial oxidative metabolism can increase luminal oxygen availability and favor facultative anaerobes, whereas microbiota-derived butyrate supports epithelial hypoxia-related signaling and barrier protection (32, 38). Whether allergen-driven type 2 inflammation produces a quantitatively meaningful change in mucosal oxygen availability sufficient to suppress obligate anaerobes or reduce SCFA production in human food allergy has not been directly established. Accordingly, the oxygenation-anaerobe-SCFA sequence is presented here as a biologically plausible hypothesis rather than an established food-allergy pathway.

### 3.3 Treg instability under allergic inflammatory pressure

Treg dysfunction may involve altered phenotype and function rather than a simple reduction in cell number. In a food-allergy mouse model, enhanced interleukin-4 (IL-4) receptor signaling promoted reprogramming of allergen-specific Tregs toward a Th2-like state and impaired oral tolerance; Th2-like features were also identified in allergen-specific Tregs from food-allergic children (39). Treg-derived TGF-β1 has additionally been shown to regulate microbiota-dependent RORγt-positive Treg differentiation and multiple checkpoints in allergic immune regulation (25), but this study does not by itself establish inflammation-induced Treg instability. Human findings are also not uniform: in one pediatric peanut study, peanut-specific Treg frequency, homing phenotype, and FOXP3 locus stability were similar across allergic and tolerant groups despite clear Th2 polarization among effector T cells (40). Thus, Th2-like Treg reprogramming is a relevant mechanistic model, but it should not be treated as a universal human phenotype or a validated determinant of SU after OIT.

### 3.4 Why desensitization may not convert into SU

OIT repeatedly delivers allergen and can reduce effector-cell reactivity while reshaping antibody and T-cell responses (5). SU requires these changes to remain clinically meaningful after treatment is stopped. A working hypothesis is that post-withdrawal durability depends not only on allergen-specific immune adaptation but also on whether the mucosal environment continues to support regulatory antigen presentation, epithelial integrity, and stable Treg function. Under this model, OIT can produce exposure-dependent protection even when the wider ecological-metabolic-immune network has not been durably altered.

Within this framework, persistent dysbiosis, inadequate local SCFA availability, incomplete epithelial repair, or unstable allergen-responsive Treg programs are candidate constraints on SU despite successful desensitization. These possibilities remain hypotheses and are not presented as established causes of failure to achieve SU.

However, this framework should not be interpreted as an established explanation for OIT failure. Clinical data already show that age, baseline IgE, dose, treatment duration, and ongoing ingestion influence outcomes (6, 7). The SCFA-Treg-gut microbiota axis is valuable because it generates measurable candidate mediators and trialable interventions, not because it displaces these established determinants. Its contribution should be tested prospectively against competing and complementary explanations. The proposed developmental and inflammatory failure modes are summarized in **Figure 3**.

**Figure 3:**
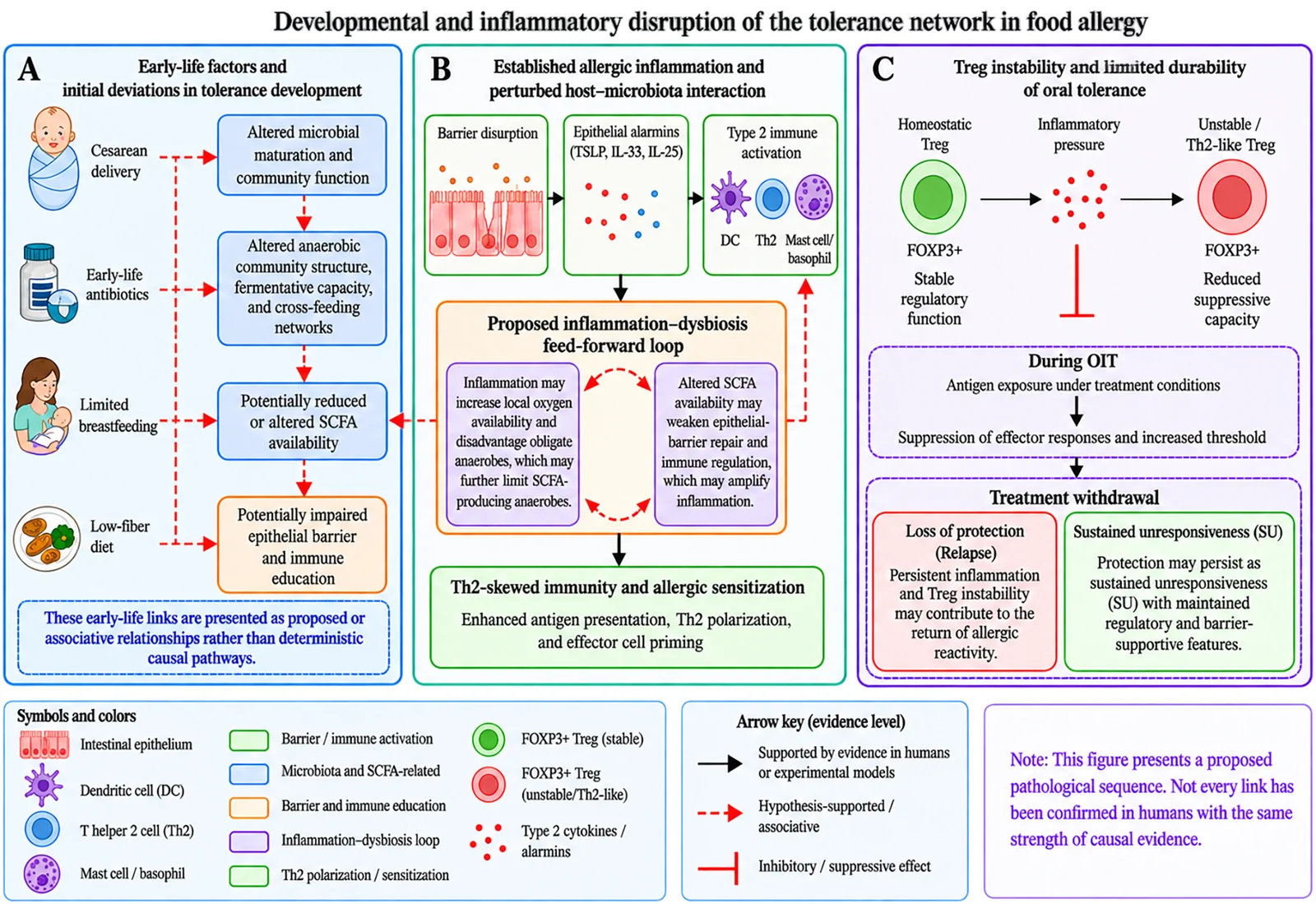
Developmental and inflammatory disruption of the tolerance network in food allergy. **(A)** Perinatal and early-life exposures, including cesarean delivery, antibiotic exposure, limited breastfeeding, and a low-fiber diet, may be associated with altered microbial maturation and community function. Potential consequences include changes in anaerobic community structure, fermentative capacity, and microbial cross-feeding, which may contribute to reduced or altered local SCFA availability. These changes could, in turn, be associated with impaired epithelial-barrier maturation and altered immune education, including changes in mucus production, goblet-cell function, tight-junction integrity, and tolerogenic signaling. These early-life links are presented as proposed or associative relationships rather than deterministic causal pathways. **(B)** In established allergic inflammation, epithelial injury and increased permeability can facilitate allergen translocation and promote epithelial alarmin signaling, including TSLP, IL-33, and IL-25, together with type 2 cytokine responses. These signals can support dendritic-cell activation, Th2 effector polarization, and mast-cell or basophil priming. A proposed inflammation–dysbiosis feed-forward loop is also shown: inflammation may increase local oxygen availability and thereby disadvantage obligate anaerobes, altered SCFA availability may weaken epithelial-barrier repair, and barrier injury, dysbiosis, and immune deviation may reinforce one another. This ecological loop remains hypothesis-generating and requires further validation in human food allergy. **(C)** Under homeostatic conditions, FOXP3-positive Tregs maintain suppressive cytokine production, tissue-adapted regulatory phenotypes, and oral tolerance. Under inflammatory pressure, exposure to type 2 and epithelial cytokines may contribute to Treg instability or Th2-like reprogramming and reduced suppressive competence. During OIT, clinical protection may depend on continued allergen exposure and the balance between effector and regulatory mechanisms. After treatment withdrawal, protection may either decline, with return of clinical reactivity, or persist as sustained unresponsiveness (SU). Solid black arrows indicate relationships supported by experimental or clinical evidence, whereas red dashed arrows indicate proposed or associative relationships requiring further validation. The figure presents a hypothesis-driven sequence integrating human associations and experimental findings; not every link has been definitively confirmed in patients.

## 4 Evidence gaps and interpretive boundaries

### 4.1 Animal causality does not establish human therapeutic efficacy

Germ-free, antibiotic-treated, and genetically modified mice allow causal manipulation of microbiota, receptors, and immune cells, but they differ from patients in diet, developmental history, microbial complexity, treatment exposure, and environmental context. Conventional laboratory mice also possess microbiota and immune phenotypes that may not reproduce human physiology; even wild-microbiota transfer can substantially change disease resistance (41). Human microbiomes show marked interindividual variation and functional redundancy (42). A successful microbial or metabolite intervention in mice therefore establishes plausibility, not expected clinical efficacy or SU benefit.

### 4.2 Fecal SCFAs are not equivalent to mucosal exposure

Fecal SCFA concentrations are affected by production, transit, epithelial consumption, absorption, and host metabolism. Stable-isotope studies demonstrate that *in vivo* production and systemic appearance cannot be inferred directly from stool abundance (19). A low fecal concentration may reflect low production or efficient uptake; a high value may reflect high production, impaired absorption, rapid transit, or altered utilization. Studies of SU should therefore combine stool and plasma metabolites with microbial pathway data, dietary intake, transit-related variables, epithelial markers, and clinical challenge outcomes. Where feasible, mucosal sampling or spatially informative surrogate measures are needed. The distinction between luminal SCFA abundance and local cellular exposure, together with selected epithelial, antigen-presenting-cell, and Treg interfaces, is illustrated in **Figure 4**.

**Figure 4:**
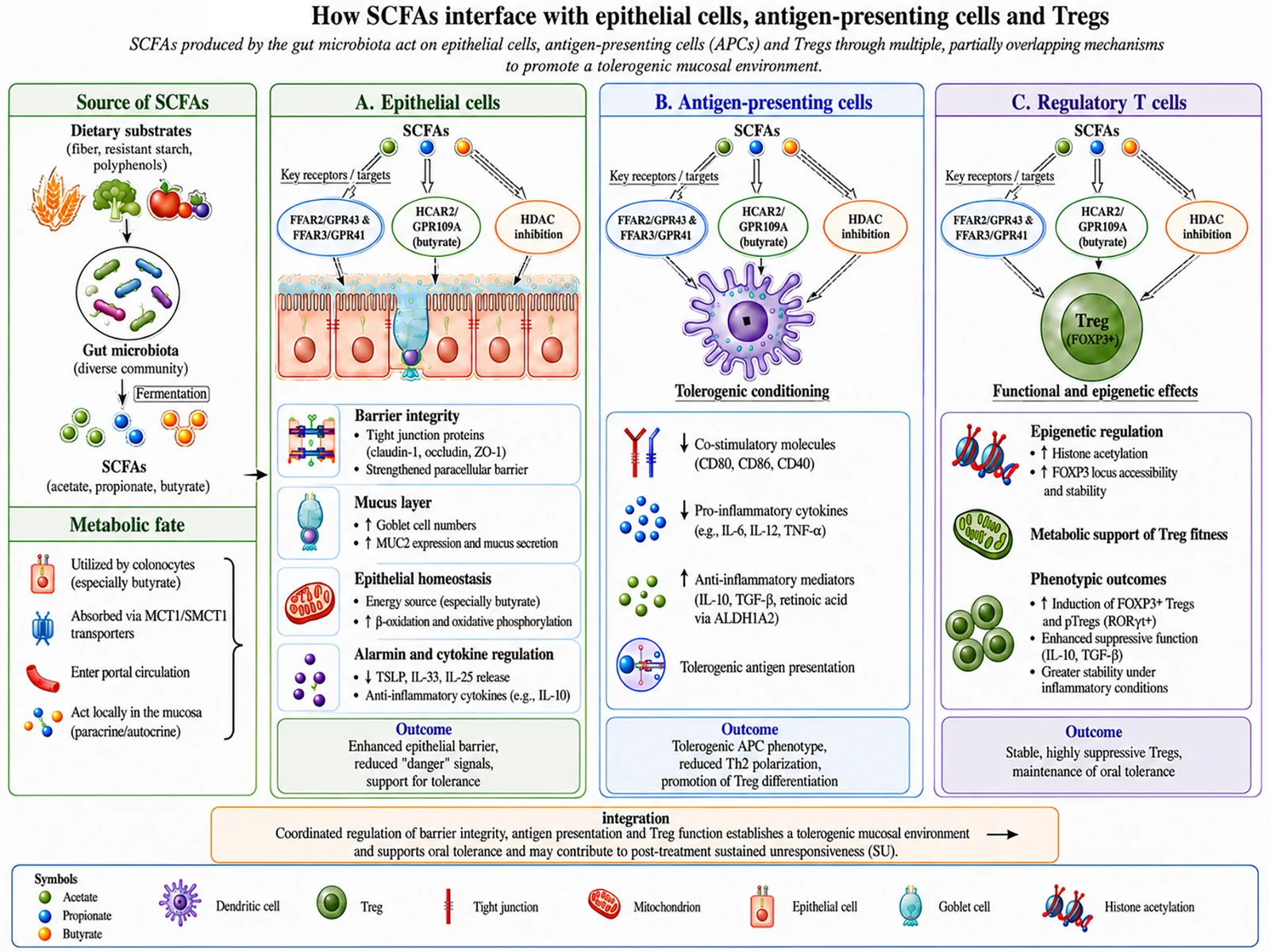
How SCFAs interface with epithelial cells, antigen-presenting cells, and Tregs. Dietary fibers, resistant starches, and other fermentable substrates are metabolized by the gut microbiota to generate the major SCFAs acetate, propionate, and butyrate. Following production, SCFAs may be utilized locally by colonocytes, absorbed through monocarboxylate transporters, enter the portal circulation, or act within the mucosal compartment. **(A)** In epithelial cells, SCFAs may act through selected metabolite-sensing receptors and intracellular targets, including FFAR pathways, HCAR2/GPR109A, and HDAC inhibition. Potential downstream effects include support of tight-junction proteins and paracellular-barrier integrity, increased mucus production and goblet-cell function, epithelial metabolic homeostasis, and modulation of epithelial alarmin and cytokine release. **(B)** In antigen-presenting cells, SCFAs may contribute to tolerogenic conditioning by reducing co-stimulatory molecules and pro-inflammatory cytokines while supporting anti-inflammatory mediators, retinoic-acid-associated pathways, and tolerogenic antigen presentation. These effects may reduce Th2 polarization and favor Treg differentiation. **(C)** In Tregs, SCFAs may promote epigenetic regulation through increased histone acetylation and enhanced accessibility and stability of the FOXP3 locus, while also supporting cellular metabolic fitness. The resulting phenotype may include increased induction of FOXP3-positive Tregs and RORγt-positive peripheral Tregs, enhanced IL-10- and TGF-β-associated suppressive function, and greater regulatory stability under inflammatory conditions. Coordinated regulation of epithelial-barrier integrity, antigen presentation, and Treg function may establish a tolerogenic mucosal environment that supports oral tolerance and could contribute to post-treatment SU. The receptors and cellular effects shown are selected examples, and their relative importance is cell-, tissue-, dose-, and context-dependent. ALDH1A2, aldehyde dehydrogenase 1 family member A2; MCT1, monocarboxylate transporter 1; MUC2, mucin 2; pTregs, peripherally induced regulatory T cells; SMCT1, sodium-coupled monocarboxylate transporter 1; ZO-1, zonula occludens-1.

### 4.3 A negative butyrate-adjuvant trial limits simple replacement models

The randomized oral peanut immunotherapy with butyrate adjuvant (OPIA) trial directly tested oral butyrate as an adjunct to peanut OIT. After the protocol-defined withdrawal period, SU occurred in 73% of the OIT-plus-butyrate group and 69% of the OIT-plus-placebo group, a non-significant difference (p=0.76) (43). This finding does not invalidate SCFA biology, but it argues against the assumption that free oral butyrate alone can reproduce endogenous fermentation, spatial delivery, cross-feeding, or cell-specific exposure. It also illustrates why mechanistic promise must be separated from intervention efficacy.

### 4.4 SU definitions and sampling schedules remain heterogeneous

Cross-trial comparison is complicated by different maintenance doses, treatment durations, avoidance intervals, challenge thresholds, and definitions of ongoing free ingestion. A short withdrawal period may capture persistence of desensitization rather than a deeply stable state, whereas longer withdrawal increases clinical relevance but also introduces adherence and exposure variability. Microbiome and immune sampling is similarly inconsistent. A minimum core outcome set should include end-of-treatment desensitization, protocol-defined SU or remission, exact avoidance duration, challenge dose, adverse events, free-ingestion behavior, and at least one longer-term post-treatment assessment.

**Table 1** compares the principal evidence domains by biological or clinical context, evidence base, and proximity to the SU endpoint, thereby distinguishing direct clinical evidence from indirect or hypothesis-generating support.

**Table 1:** Evidence hierarchy for the SCFA-Treg-gut microbiota framework across food allergy and OIT outcomes.

Domain/clinical context	Key evidence/evidence base	Relevance to SU	Interpretation/limitation/refs
Clinical OIT outcomes. OIT desensitization and post-withdrawal outcomes	Randomized trials show robust desensitization but lower or heterogeneous remission/SU. Human randomized trials.	Direct	SU is clinically achievable, but definitions, withdrawal intervals, treatment duration, and ongoing ingestion vary across trials. Refs (6, 7, 43, 44):
SCFA-Treg mechanisms. Regulatory immunity and oral tolerance	SCFAs regulate Tregs through HDAC inhibition and metabolite-sensing receptors. Predominantly mouse and *in vitro* mechanistic evidence.	Indirect	Strong biological plausibility; direct evidence linking these mechanisms to post-OIT SU in humans remains limited. Refs (11–13, 20–24, 26–28):
Fiber and microbial metabolism. Community fermentative capacity	Substrate use, pathway distribution, and microbial cross-feeding shape SCFA output. Human gut isolates and mechanistic/microbiome studies.	Indirect	Taxonomy alone is an incomplete proxy for metabolic function; functional capacity and spatial exposure are more relevant. Refs (10, 17, 18):
Food-allergy models. Sensitization and oral tolerance	Dietary fiber/SCFA pathways, tolerogenic APC programs, and epithelial alarmins alter allergic responses in mouse food-allergy models.	Indirect	Directly relevant to food-allergy biology, but does not establish durability after OIT withdrawal. Refs (29, 31, 39):
Early-life human microbiota. Food sensitization and allergy risk	Longitudinal cohorts associate microbial maturation and SCFA-related features with later allergic outcomes; human-to-mouse transfer adds experimental support.	Indirect	Developmental relevance is supported, but signatures are heterogeneous and susceptible to confounding. Refs (15, 33–37):
Direct metabolite supplementation. OIT to SU	OPIA tested oral butyrate as an OIT adjunct and did not improve protocol-defined SU. Human randomized trial.	Direct	Free oral butyrate was not sufficient to improve SU in the OPIA trial; this does not exclude effects of other formulations or delivery strategies. Refs (43):
Microbiota-based interventions. Food-allergy treatment	FMT, defined microbial consortia, targeted delivery, prebiotics, synbiotics, and probiotics range from preclinical platforms to early clinical studies.	Limited direct evidence	Safety, engraftment, function, spatial delivery, and efficacy specifically for SU remain unresolved. Refs (44–51):

## 5 Translational strategies

Several microbiota- and metabolite-directed strategies are being investigated as adjuncts to antigen-specific therapy, with markedly different levels of preclinical and clinical evidence. Their relative translational maturity is summarized in **Figure 5**.

**Figure 5:**
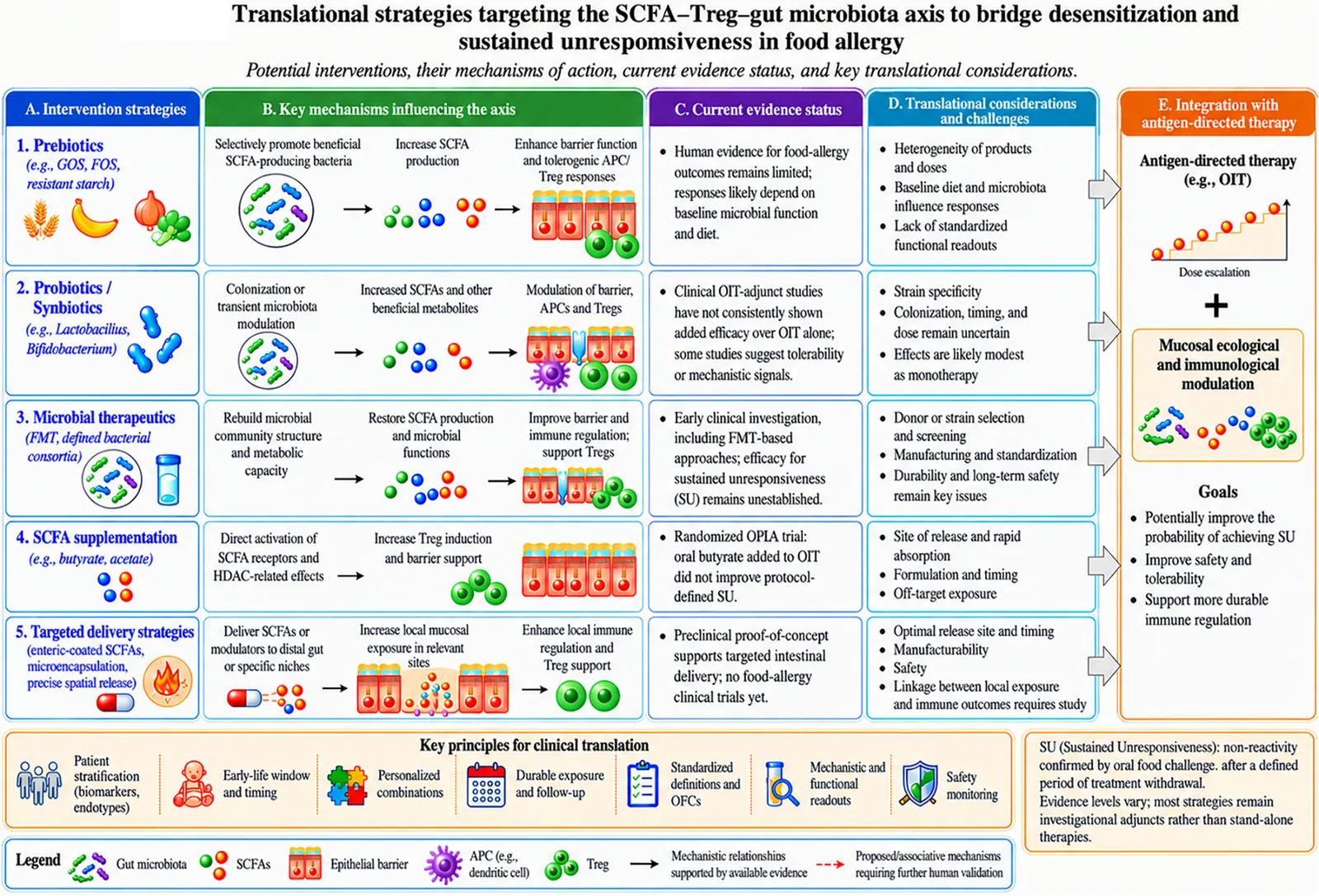
Translational strategies targeting the SCFA–Treg–gut microbiota axis to bridge desensitization and sustained unresponsiveness in food allergy. **(A)** Candidate intervention strategies include prebiotics, probiotics or synbiotics, microbial therapeutics such as fecal microbiota transplantation (FMT) or defined bacterial consortia, direct SCFA supplementation, and targeted intestinal delivery strategies. **(B)** Although these approaches differ in composition and mode of administration, their proposed mechanisms converge on modulation of microbial community function, increased or restored SCFA and beneficial-metabolite production, reinforcement of epithelial-barrier function, tolerogenic conditioning of antigen-presenting cells, and support of Treg induction and function. Targeted-delivery approaches additionally aim to increase metabolite exposure at relevant intestinal sites. **(C)** The current evidence supporting these strategies differs substantially in maturity. Human evidence for prebiotic effects on food-allergy outcomes remains limited. Probiotic or synbiotic adjuncts to OIT have not consistently demonstrated additional efficacy over OIT alone, although mechanistic or tolerability signals have been reported. Microbial therapeutics, including FMT-based approaches, remain in early clinical investigation and have not established efficacy for SU. In the randomized OPIA trial, oral butyrate added to OIT did not improve protocol-defined SU. Targeted intestinal SCFA delivery currently remains supported primarily by preclinical proof-of-concept, without established food-allergy clinical efficacy. **(D)** Major translational challenges include product and dose heterogeneity, dependence on baseline diet and microbiota, strain or donor selection, colonization and engraftment, manufacturing and standardization, formulation and site of release, durability, linkage between local metabolite exposure and immune outcomes, and long-term safety. **(E)** A proposed translational framework combines antigen-directed therapy, such as OIT, with mucosal ecological and immunological modulation. The goals of such combinations are to test whether they can increase the probability of achieving SU, improve safety or tolerability, and support more durable immune regulation rather than to assume established clinical benefit. Key principles for future translation include patient stratification, attention to developmental timing, personalized intervention combinations, adequate exposure and longitudinal follow-up, standardized definitions and oral food challenges, mechanistic and functional readouts, and safety monitoring. Solid arrows indicate mechanistic relationships supported by available evidence, whereas dashed arrows indicate proposed or associative mechanisms requiring further human validation. SU denotes clinical non-reactivity confirmed by oral food challenge after a defined period of treatment withdrawal; most microbiota- or metabolite-directed strategies remain investigational adjuncts rather than established stand-alone therapies. FOS, fructo-oligosaccharides; GOS, galacto-oligosaccharides; OFC, oral food challenge.

### 5.1 Fecal microbiota transplantation and defined microbial therapeutics

Oral encapsulated fecal microbiota transplantation (FMT) has entered early clinical investigation in peanut allergy, including a phase I study and a registered phase II study (45, 46). These programs show that microbiota-directed treatment has moved beyond mechanistic speculation, but efficacy for SU has not been established. FMT also introduces donor variability, uncertain engraftment, and the possibility of transmitting pathogenic or antimicrobial-resistant organisms. The US Food and Drug Administration has issued safety alerts concerning serious infections associated with investigational FMT (47).

For food allergy, defined microbial therapeutics may ultimately offer greater control than crude FMT because composition, potency, contaminants, and manufacturing can be standardized. However, strain inclusion should be based on reproducible function rather than taxonomic reputation alone. Required development steps include genomic safety assessment, metabolite and potency assays, product stability, engraftment monitoring, and long-term surveillance (48).

### 5.2 Spatially targeted SCFA delivery

Free oral butyrate has unfavorable taste and odor and may be absorbed before reaching the intended intestinal compartment. Polymeric micelles that release butyrate in different gut regions reduced allergic responses and improved barrier-related measures in mouse models (49). The conceptual advance is spatial pharmacology: a metabolite may be effective only when delivered at an adequate concentration, for an adequate duration, to the cells and microbial communities that can use it. Translation requires human data on release reproducibility, material safety, exposure biomarkers, dosing schedules, and clinically meaningful endpoints.

### 5.3 Precision prebiotics, synbiotics, and probiotic adjuncts

Prebiotic interventions should match substrate to microbial capacity rather than assume that generic fiber supplementation will increase butyrate in every patient. A synbiotic combining Anaerostipes caccae with lactulose increased luminal butyrate and prevented or treated food allergy in mice (50). This provides proof of principle for substrate-strain matching, but remains preclinical and does not establish engraftment or efficacy in patients.

Clinical probiotic-OIT data also require careful interpretation. The initial randomized probiotic-peanut OIT study showed a high SU rate for the combination but lacked an OIT-only arm, so the probiotic contribution could not be isolated (51). PPOIT-003 subsequently compared probiotic plus OIT, OIT alone, and placebo; adding the probiotic did not improve efficacy over OIT alone, although tolerability signals were reported in younger children (44). These findings argue for functionally selected strains, prespecified microbial mechanisms, and direct comparison with the antigen-specific treatment alone.

### 5.4 Biomarker-guided combination trials

A testable strategy is to combine antigen-directed therapy with ecological or metabolic intervention in a staged design. Baseline profiling could identify patients with low microbial SCFA-production capacity, barrier dysfunction, or unstable regulatory phenotypes. An ecological intervention could then be tested before OIT, during maintenance, or around withdrawal, with randomization determining whether timing changes SU. This proposal should be framed as a trial design hypothesis rather than a current treatment recommendation.

Future studies should integrate dietary records, metagenomic or metatranscriptomic pathway data, stool and plasma metabolites, epithelial-barrier markers, allergen-specific IgE/IgG4, basophil activation, and longitudinal T-cell phenotypes. Public microbiome and multi-omics frameworks demonstrate the feasibility of standardized data integration (52, 53), but food-allergy studies need allergen-specific sampling and post-withdrawal clinical endpoints. Composite prediction models should be developed in training cohorts and validated prospectively rather than inferred from small, high-dimensional datasets.

## 6 Conclusions

The therapeutic objective in food allergy is moving beyond exposure-dependent desensitization toward durable post-treatment immune control. The SCFA-Treg-gut microbiota axis offers a coherent framework for investigating this transition because it connects microbial fermentation with regulatory immunity, epithelial function, and antigen presentation. Its strongest causal evidence, however, comes from experimental models, and human associations do not yet establish that modifying this axis will increase SU.

The negative OPIA result is particularly informative: biologically active metabolites cannot be assumed to work as simple oral replacements. Translational studies should therefore prioritize spatial delivery, defined microbial function, harmonized SU definitions, and biomarker-guided randomized designs. Microbiota or metabolite interventions should be evaluated as adjuncts to antigen-directed therapy, not presented as established substitutes. With this evidence hierarchy, the axis can serve as a productive, falsifiable model for understanding why some patients retain protection after OIT withdrawal while others do not (14).
